# Supplementary material for: Pyruvate Dehydrogenase Kinase 1 inhibition mediated oxidative phosphorylation enhancement in cartilage promotes osteoarthritis progression
Source: BMC Musculoskelet Disord. 2023 Jul 20;24:597. doi: 10.1186/s12891-023-06585-6 (PMC10357736; doi:10.1186/s12891-023-06585-6)
Supplement: Supplementary file 1 — Additional file 1. [file 12891_2023_6585_MOESM1_ESM.zip › MF.Gsea.1653627316242/GOMF_CARBOXYPEPTIDASE_ACTIVITY.html]

Details for gene set GOMF\_CARBOXYPEPTIDASE\_ACTIVITY[GSEA]

|  || Dataset | 55457\_collapsed\_to\_symbols.cls.cls #OA\_versus\_normal.cls.cls #OA\_versus\_normal\_repos |
| Phenotype | cls.cls#OA\_versus\_normal\_repos |
| Upregulated in class | OA |
| GeneSet | GOMF\_CARBOXYPEPTIDASE\_ACTIVITY |
| Enrichment Score (ES) | 0.4466194 |
| Normalized Enrichment Score (NES) | 1.6497121 |
| Nominal p-value | 0.014705882 |
| FDR q-value | 0.12106367 |
| FWER p-Value | 0.937 |
Table: GSEA Results Summary

  

Fig 1: Enrichment plot: GOMF\_CARBOXYPEPTIDASE\_ACTIVITY      
 Profile of the Running ES Score & Positions of GeneSet Members on the Rank Ordered List

  

| SYMBOL | TITLE | RANK IN GENE LIST | RANK METRIC SCORE | RUNNING ES | CORE ENRICHMENT || 1 | PEPD | peptidase D [Source:HGNC Symbol;Acc:HGNC:8840] | 167 | 0.842 | 0.0728 | Yes |
| 2 | CPQ | carboxypeptidase Q [Source:HGNC Symbol;Acc:HGNC:16910] | 257 | 0.780 | 0.1452 | Yes |
| 3 | CPVL | carboxypeptidase vitellogenic like [Source:HGNC Symbol;Acc:HGNC:14399] | 459 | 0.677 | 0.1986 | Yes |
| 4 | AGBL3 | AGBL carboxypeptidase 3 [Source:HGNC Symbol;Acc:HGNC:27981] | 593 | 0.626 | 0.2520 | Yes |
| 5 | MINDY2 | MINDY lysine 48 deubiquitinase 2 [Source:HGNC Symbol;Acc:HGNC:26954] | 863 | 0.552 | 0.2873 | Yes |
| 6 | CPA3 | carboxypeptidase A3 [Source:HGNC Symbol;Acc:HGNC:2298] | 1259 | 0.465 | 0.3040 | Yes |
| 7 | CNDP2 | carnosine dipeptidase 2 [Source:HGNC Symbol;Acc:HGNC:24437] | 1476 | 0.432 | 0.3312 | Yes |
| 8 | SCPEP1 | serine carboxypeptidase 1 [Source:HGNC Symbol;Acc:HGNC:29507] | 1520 | 0.426 | 0.3713 | Yes |
| 9 | CPE | carboxypeptidase E [Source:HGNC Symbol;Acc:HGNC:2303] | 1796 | 0.385 | 0.3891 | Yes |
| 10 | CPB1 | carboxypeptidase B1 [Source:HGNC Symbol;Acc:HGNC:2299] | 1918 | 0.370 | 0.4174 | Yes |
| 11 | CTSA | cathepsin A [Source:HGNC Symbol;Acc:HGNC:9251] | 2014 | 0.360 | 0.4466 | Yes |
| 12 | AGBL5 | AGBL carboxypeptidase 5 [Source:HGNC Symbol;Acc:HGNC:26147] | 2613 | 0.290 | 0.4296 | No |
| 13 | LAP3 | leucine aminopeptidase 3 [Source:HGNC Symbol;Acc:HGNC:18449] | 3044 | 0.245 | 0.4212 | No |
| 14 | CPZ | carboxypeptidase Z [Source:HGNC Symbol;Acc:HGNC:2333] | 4043 | 0.152 | 0.3591 | No |
| 15 | VASH2 | vasohibin 2 [Source:HGNC Symbol;Acc:HGNC:25723] | 4345 | 0.124 | 0.3484 | No |
| 16 | NAALAD2 | N-acetylated alpha-linked acidic dipeptidase 2 [Source:HGNC Symbol;Acc:HGNC:14526] | 4870 | 0.082 | 0.3161 | No |
| 17 | CPB2 | carboxypeptidase B2 [Source:HGNC Symbol;Acc:HGNC:2300] | 5279 | 0.050 | 0.2894 | No |
| 18 | NAALADL1 | N-acetylated alpha-linked acidic dipeptidase like 1 [Source:HGNC Symbol;Acc:HGNC:23536] | 6024 | -0.006 | 0.2322 | No |
| 19 | MINDY1 | MINDY lysine 48 deubiquitinase 1 [Source:HGNC Symbol;Acc:HGNC:25648] | 6126 | -0.014 | 0.2258 | No |
| 20 | CTSZ | cathepsin Z [Source:HGNC Symbol;Acc:HGNC:2547] | 6292 | -0.027 | 0.2157 | No |
| 21 | PRCP | prolylcarboxypeptidase [Source:HGNC Symbol;Acc:HGNC:9344] | 6725 | -0.057 | 0.1880 | No |
| 22 | AEBP1 | AE binding protein 1 [Source:HGNC Symbol;Acc:HGNC:303] | 6822 | -0.065 | 0.1871 | No |
| 23 | ACE2 | angiotensin converting enzyme 2 [Source:HGNC Symbol;Acc:HGNC:13557] | 6951 | -0.076 | 0.1849 | No |
| 24 | FOLH1 | folate hydrolase 1 [Source:HGNC Symbol;Acc:HGNC:3788] | 7048 | -0.083 | 0.1859 | No |
| 25 | CPD | carboxypeptidase D [Source:HGNC Symbol;Acc:HGNC:2301] | 7433 | -0.114 | 0.1676 | No |
| 26 | CPM | carboxypeptidase M [Source:HGNC Symbol;Acc:HGNC:2311] | 8199 | -0.171 | 0.1256 | No |
| 27 | BLMH | bleomycin hydrolase [Source:HGNC Symbol;Acc:HGNC:1059] | 8219 | -0.173 | 0.1417 | No |
| 28 | VASH1 | vasohibin 1 [Source:HGNC Symbol;Acc:HGNC:19964] | 8473 | -0.191 | 0.1415 | No |
| 29 | CPN1 | carboxypeptidase N subunit 1 [Source:HGNC Symbol;Acc:HGNC:2312] | 8613 | -0.202 | 0.1513 | No |
| 30 | AGBL2 | AGBL carboxypeptidase 2 [Source:HGNC Symbol;Acc:HGNC:26296] | 9559 | -0.279 | 0.1063 | No |
| 31 | ACE | angiotensin I converting enzyme [Source:HGNC Symbol;Acc:HGNC:2707] | 9720 | -0.294 | 0.1237 | No |
| 32 | CPA2 | carboxypeptidase A2 [Source:HGNC Symbol;Acc:HGNC:2297] | 9774 | -0.298 | 0.1500 | No |
| 33 | AGTPBP1 | ATP/GTP binding carboxypeptidase 1 [Source:HGNC Symbol;Acc:HGNC:17258] | 10752 | -0.399 | 0.1147 | No |
| 34 | CPA1 | carboxypeptidase A1 [Source:HGNC Symbol;Acc:HGNC:2296] | 11581 | -0.512 | 0.1025 | No |
Table: GSEA details [plain text format]

  

Fig 2: GOMF\_CARBOXYPEPTIDASE\_ACTIVITY      
 Blue-Pink O' Gram in the Space of the Analyzed GeneSet

  

Fig 3: GOMF\_CARBOXYPEPTIDASE\_ACTIVITY: Random ES distribution      
 Gene set null distribution of ES for **GOMF\_CARBOXYPEPTIDASE\_ACTIVITY**
